# Why women chose unassisted home birth in Malaysia: a qualitative study

**DOI:** 10.1186/s12884-020-02987-9

**Published:** 2020-05-19

**Authors:** Nur Amani Natasha Ahmad Tajuddin, Julia Suhaimi, Siti Nurkamilla Ramdzan, Khasnur Abd Malek, Ilham Ameera Ismail, Nurainul Hana Shamsuddin, Ahmad Ihsan Abu Bakar, Sajaratulnisah Othman

**Affiliations:** 1 Department of Primary Care Medicine, Faculty of Medicine, University of Malaya, Kuala Lumpur, Malaysia; 2 Department of Primary Care Medicine, Faculty of Medicine, Universiti Teknologi MARA, Selangor, Malaysia; 3 Department of Family Medicine, Faculty of Medicine & Health Sciences, Universiti Putra Malaysia, Serdang, Malaysia

**Keywords:** Homebirth, Free birth, Unassisted home birth, Malaysia, Healthcare delivery system, Qualitative

## Abstract

**Background:**

Incidences of unassisted home birthing practices have been increasing in Malaysia despite the accessibility to safe and affordable child birthing facilities. We aimed to explore the reasons for women to make such decisions.

**Methods:**

Twelve women participated in in-depth interviews. They were recruited using a snowballing approach. The interviews were supported by a topic guide which was developed based on the Theory of Planned Behaviour and previous literature. The interviews were audio-recorded, transcribed verbatim and analysed using thematic analysis.

**Results:**

Women in this study described a range of birthing experiences and personal beliefs as to why they chose unassisted home birth. Four themes emerged from the interviews; i) preferred birthing experience, ii) birth is a natural process, iii) expressing autonomy and iv) faith. Such decision to birth at home unassisted was firm and steadfast despite the possible risks and complications that can occur. Giving birth is perceived to occur naturally regardless of assistance, and unassisted home birth provides the preferred environment which health facilities in Malaysia may lack. They believed that they were in control of the birth processes apart from fulfilling the spiritual beliefs.

**Conclusions:**

Women may choose unassisted home birth to express their personal views and values, at the expense of the health risks. Apart from increasing mothers’ awareness of the possible complications arising from unassisted home births, urgent efforts are needed to provide better birth experiences in healthcare facilities that resonate with the mothers’ beliefs and values.

## Background

The World Health Organisation (WHO) aims to reduce maternal and neonatal mortality in all countries by 2030 as one of the targets for the Sustainable Development Goals 3 (SDG 3) [1]. The presence of skilled health personnel during births is crucial to achieving this aim, and the proportion of births attended by skilled health personnel is one of the critical indicators used to monitor the achievement of the goal [1]. To ensure this, WHO has refined the definition of skilled health personnel providing care for childbirth as ‘competent maternal and new-born health professionals educated, trained and regulated to national and international standards’ [2]. Most of the births attended by skilled health personnel occur at healthcare facilities.

However, some women prefer birthing at home, as they believe it is safer and provides a better outcome for the mother and baby as compared to birthing at a hospital [3]. In the Netherlands, women with low obstetric risk are given a choice to either birth at home under the supervision of a community midwife or in the hospital [4]. Based on the 2018 perinatal registry in the Netherlands, the percentages of births at home, assisted by community midwife or general practitioner was 12.9%.

Planning for birth at home based on risk stratification is also practised in some high-income countries such as the United Kingdom and Australia. These births are planned and only offered to women with uncomplicated pregnancies [5, 6]. These are made possible with the availability of trained and skilled health personnel to attend to births at home [1]. However, in low and middle-income countries, home births are usually not attended by skilled health professionals due to the lack of workforce and resources [1]. Limited access to healthcare and the costs of birthing in the hospital are also the reasons why women are resorting to birthing at home in low- and middle-income countries [7].

While the role of skilled health personnel for assisted home births is well established, there is a trend of mothers employing “doulas” or birth companions for their unassisted home births. A doula is a woman who is trained and experienced in childbirth and provides continuous physical, emotional, and informational support to a woman during labour, birth, and the immediate postpartum period [8]. The doulas are expected to be present throughout the birth process, whether it is at home or hospital. Several organizations of doulas have explicitly detailed the role of a doula, which is to provide emotional support, and physical comfort to a mother before, during and just after childbirth [9, 10]. It does not include giving medical advice and any decision-making about the health of mother and baby.

Women choose home birth without skilled birth attendance for various reasons. Physical distance and financial limitation were the two significant constraints in Indonesia [11]. While in Laos, women and their husbands perceived greater advantages of home birth, including convenience, time and able to be near to family with home birth compared to hospital births [12]. In Australia, despite the access to having skilled health personnel to attend births at home, some women maintain to opt for unassisted home births or free birth. These women regarded the mainstream system as inflexible as they were not able to access the birth centre of their choice, and the guidelines and criteria are prohibitive to them. They also viewed their previous birth in the system as traumatising [13].

Historically, home birthing in Malaysia was conducted by traditional birth attendants (TBAs) who learned from experience and knowledge passed down from one generation to the other [14]. These traditional birth attendants are not healthcare personnel. The government later introduced formal training to these traditional birth attendants that resulted in a dramatic reduction in maternal and infant mortality rate of the country [14]. Subsequently, all nurses in the community health clinics were trained to conduct births with a proportion of them receiving further post-basic training in midwifery. A pathway for assisted home births has existed for low-risk mothers for many years [15]. However, this practice is rarely carried out due to the current work burden faced by these community nurses within the hospital and community health clinic setting. Their tight schedule involves various clinical duties, including home visits, maternal and child healthcare clinic, and other primary health care duties clinics. With limited human resource and financial input, comprehensive care towards safe handling of assisted home birth is still far reach. However, a low-risk maternity centre was set up in Putrajaya, Malaysia in 2012 to meet the need for mothers who wish to deliver in a setting that is closest to resembling a home setting. This centre was set up to almost resemble birth at home, with fully equipped medical facilities, at a little cost to cater for the locals and at a reasonable rate for foreigners [16]. Other options for births are in the hospitals (government or private) settings.

In Malaysia, there has been a drastic reduction in the rate of maternal mortality, from 540 deaths per 100,000 births in the 1950s to 28 deaths in 100,000 births in 2016. This reduction is observed following a drastic improvement in access to health services and universal health coverage. However, there has been an increasing trend of pregnancy-related deaths outside the health facilities over the past decade. There was an increase of homebirth deaths; unassisted birth or attended by an unskilled person, from 7 in 2008 to 13 in 2014 [17, 18]. This increase is worrying, especially in the local setting, where unassisted home birth has been in increasing popularity, resulting in the death of the mother and the baby.

Newspapers have reported that women took the risk to give birth at home without the presence of skilled health personnel when their wishes to have home birth were turned down by health care personnel. They resorted to home birth support groups to obtain confidence and support to give birth at home [19]. The extensive use of social media and access to various information through the internet may have played a role in encouraging the growth and influence of these groups. Some mothers who had a pleasant unassisted home birthing experience uploaded their birth stories online, and this could give false positive reassurance to other mothers that are thinking about doing the same [20].

To the best of our knowledge, there has not been any published literature on reasons why women choose unassisted home birth in Malaysia. Our study aims to explore the reasons why women choose unassisted home births in Malaysia.

## Methods

### Design

We used a qualitative approach to explore women’s views and perception of their decision for an unassisted home birth. This approach enables a deeper understanding of their perceptions, perspectives and understandings on the phenomenon [21]. The interviews were framed into three phases; reconstructing recent experience in unassisted home birth, detailing the experience and reflecting on the experiences [22]. The topic guide was developed based on the theory of planned behaviour and literature findings on the reasons for choosing home birth (Table 1). Based on this theory, the behaviour in focus (in this study, which is having an unassisted home birth) is associated with the person’s intention and this intention is influenced by the three domains; attitude, subjective norm and perceived ability [23]. This study received medical ethics clearance from the Medical Ethics Committee of University Malaya Medical Centre (MREC ID: 201591634). All the participants consented to participate in this study.

**Table 1:** Semi-structured topic guide

Interview topic guide	
OPENING	
1. We are exploring the decision on unassisted home birthing. Can you tell us about your birth experience?	
2. Do you want to explain your home birthing?	
DECISION ON UNASSISTED HOME BIRTHING	
3. Describe how you would like to give birth?	
4. Can you describe your ideal environment during labour?	
5. Where is the ideal place for you to give birth?	
6. What are your considerations when choosing a birthing place?	
7. What is the most important consideration in choosing a birthing place?	
8. Who decides or influence your choice of birthing place?	
9. How do you decide on your choice?	
10. Do you have any fear when considering a birthing place?	
RISKS	
11. Any disadvantage(s)/risk(s) on the birthplace of your choice?	
12. How do you overcome this advantage(s)/risk(s)?	
TIMING	
13. When do you decide on your birthplace?	
14. Would you consider changing your decision about the birthing place? When? Why?	

### Setting

This study was conducted in Kuala Lumpur, the capital city of Malaysia, where health facilities including hospitals (public and private) are easily accessible. According to the Malaysia National Health Morbidity Survey 2015, the mean distance to the government health clinic is about 9.8 km [24]. Access to health care services can be obtained either from the public or private sector, with the public sector being heavily subsidized by the government to allow easy access to all socioeconomic groups [25].

### Recruitment and data collection

Purposive sampling was used to recruit women who were above 18 years old with intentional unassisted home birth experience. We used a snowballing method to recruit the women as it is suitable to find an unattainable population [26]. We initially approached a key person via the social media of a local unassisted homebirth support group; who later introduced us to other potential participants. Written consent was obtained from women who agreed to participate.

The women were interviewed individually using a semi-structured topic guide [22]. We conducted in-depth interviews (IDI) as it allowed them to express their experiences in detail and voice their views openly regarding their unassisted home birth experiences, which they may otherwise not reveal in the presence of others [27]. All interviews were audio-recorded by digital audio recorders and transcribed verbatim for analysis. Identifiers were removed to ensure anonymity. Interviews lasted up to 90 min.

#### Data analysis

This study was conducted using a semi-structured topic guide based on the theory of planned behaviour. Thematic analysis was conducted in an iterative manner and started during the data collection [22]. NAAT, SNR, KA and NHS interviewed the study participants. The authors met up to discuss the first transcript and the coding frame. The subsequent transcripts were coded using the agreed coding frame, and any emerging new codes were discussed and added at each meeting. The coding for the rest of the transcripts was conducted by NAAT, SNR, KA, IAI and JS.

**Fig. 1:**
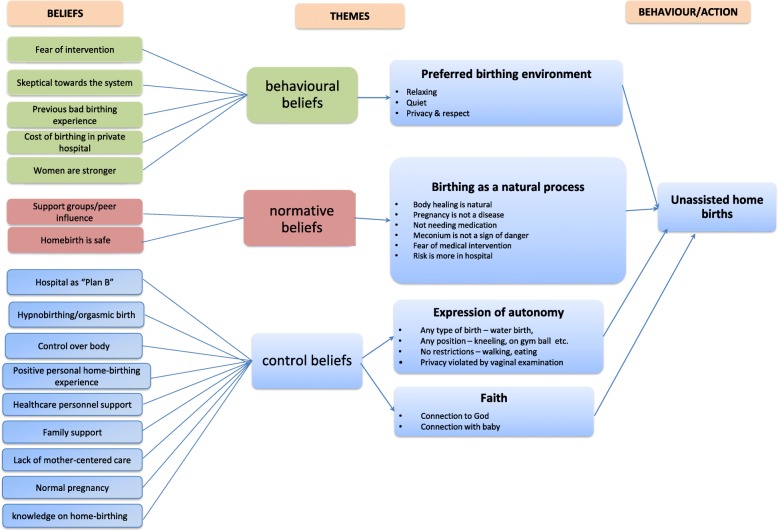
Coding framework

A deductive approach was used to link generated coding to the theory of planned behaviour. Following this, the researchers used an inductive approach to generate categories and themes. The initial codes which shared similar meanings were collated and categorized together. Categories with similar meanings were further collapsed together into meaningful themes. Overlapping themes were combined and grouped into overarching themes. NAAT, JS, SNR, AIAB, KA, IAI, NHS and SO met up to identify the codes throughout each transcript. Figure 1 describes the coding framework.

Any discrepancies were discussed, and disagreements were resolved. Data reached saturation by the 10th interview when no new theme emerged, and interviews were stopped after the 12th interview when saturation has been confirmed. All authors contributed in writing the manuscript. All authors read and approve the final manuscript.

#### Reflexivity and interpretation

The authors are all primary care physicians in public universities in Malaysia. We believe that birthing should be conducted in a manner that is clinically safe to protect the health and life of both mothers and the new-born. The findings described in this study reflect our personal perspectives and our biases as individuals, mothers and primary care doctors, which we may or may not be conscious of. Finally, we acknowledged the challenges in fully capturing the meanings of the interactions during the interview sessions, which may not be reflected in the written word.

## Results

### Participant characteristics

In total, 17 women who had unassisted home birth were approached to participate in this study. There were a few women who refused participation. Although the snowballing method was used and a few potential participants were suggested, we assumed that the potential fear of backlash from their information-sharing hindered them from becoming participants or they may have an unfavourable outcome from unassisted home birth. The authors have tried their best to invite potential participants.

Table 2 summarises the women demographic characteristics. The women’ mean age was 40.25 years +/− 3.31. All except for one participant had tertiary educational level. These women have a variable demographic background. Some were full-time housewives, childbirth advocates, and natural birth advocates. All women had their last birth unassisted at home, and one participant was a primigravida. Seven participants had one unassisted home birth, four participants had two unassisted home births, and one participant had three unassisted home births. None were pregnant at the time of the interview.

**Table 2:** Demographic characteristics of the participants (*n* = 12)

IDI code	Age range (years)	Highest academic qualification	Currently pregnant	Parity	Number of home births
1	35–39	Masters	No	2	1
2	40–44	Degree	No	3	1
3	40–44	PhD	No	3	2
4	35–39	Masters	No	1^+ 1^	1
5	35–39	Degree	No	2	1
6	40–44	Secondary school	No	3	2
7	45–49	Degree	No	7	2
8	35–39	Degree	No	3	1
9	40–44	Degree	No	4	2
10	45–49	PhD	No	7	1
11	40–44	Degree	No	1	1
12	35–39	Degree	No	3	3

### Themes

In exploring the reasons for choosing unassisted home birth, four themes emerged from the interviews; being able to have their preferred birthing environment, the belief that birthing itself is a natural and safe process, expression of autonomy and faith.

#### Theme 1: Preferred birthing environment

Women in this study repeatedly highlighted the value of home births, for it provided them with the comfort of home and the much-needed privacy during birthing. For some, perceived instrumental and chemical intervention within the hospital setting steered them towards unassisted home birth.

#### Comfort and privacy

For some women, birthing was described as a very personal and intimate process, almost mirroring the act of lovemaking. They perceived unassisted home birth as a conduit to the privacy and comfort that they aspired for. All women unanimously indicated the importance of a comfortable environment during delivery to attain their intimate needs. This became an important influencing factor in choosing home birth.

*“The birthing place; where the mother feels most safe, most comfortable, most secure would be at home and birth is a very personal, very intimate act”***IDI 1**

*“It’s (birth) usually is not to be observed. It’s just like lovemaking, it is very intimate, it’s not meant to be observed (by others)”***IDI 6**

#### Shunning stressful hospital environment

Their need for intimacy shaped their delivery preference for home-based over hospital setting. The hospital environment was described as a stressful, cold and uncomfortable place to experience the birth of their child. They felt unsafe, scared and had difficulty being at ease when they had their previous births at the hospital.

*“I want a place that I can feel safe and comfortable. I can relax and give birth easily. That’s how I gave birth at home easily. I’m affected easily by noise and hospital environment. I am very scared of the hospital environment. So I know if I couldn’t give birth at the hospital, I need to get ready to give birth at home. The sterile environment of the hospital...there is a lot of needles, sharp instruments, glaring lights. Those are very intimidating to me. I feel very insecure. When I go to the hospital, I feel like …” God, I’m going to die “ So I don’t like it”***IDI 4**

*“...so once I get into the labour room, I feel scared and stressed. It’s going to be cold. I fear the cold... it’s too cold. I’ve been telling people that I was in pain, but I have to deal with the coldness of the labour room. And then even the steel bar that I have to hold on to during pushing, it’s too cold.”***IDI 10**

Some women were traumatised by their previous experience of birthing in the hospital and this experience had very much influenced their decision to seek a comfortable environment for their subsequent delivery.

*“There must be a different way. I’m not going to go through that (hospital birth) again*.*.. all I remember was it’s not a very good experience...because I didn’t know what to expect. I didn’t really read up so I just trusted the doctor...you are like most probably dehydrated and so hungry and cold and I am just lying down there on my side …*. *it was really painful. Now I talk about it and I cringe. I had such a huge cut, and going to toilet is like.. It was horrible, horrible”***IDI 8**

*“I was admitted at 8.00 am. 8.00 am until 7.00 pm, I didn’t get to drink even one glass of water, did not eat. I was so restless and tired and was just lying on my back. It wasn’t really a labour ward because it was full and I was like in an extra room with people walking by and going in and out”***IDI 2**

### Theme 2: Birth as a natural process

#### Exposure to unassisted home birth concept

These women were initially introduced to the concept of unassisted home birth by either a close relative, friends or acquaintance. Further reading from home birth books, magazines and online materials that featured topics such as ‘painless childbirth’ or ‘hypnobirthing’ further convinced them regarding the positive side of home birthing and the ability to perform this. Following this, they went through an active phase of learning and information gathering to familiarize themselves with the delivery practices and routines. They received support by attending home birth group classes, online networking and information from online materials or books. The knowledge and support helped to solidify and further strengthened their decision making for a home birth.

*“... this yoga teacher educated us on the process of giving birth-what happens during your caesarean (section). And then, the mother who just gave birth naturally at home shared with me what happened and things like that. So, I’m like “Oh, pretty interesting eh?” so that’s how I shifted my mind.”***IDI 12**

*“I wanted to know more so I read more about pregnancy and all that. So when I read about that hypnobirthing...it just say “this is what I want”. The article is really good. It’s about removing fear, the joy of being pregnant, the joy of giving birth. So, I looked up to the internet, found out more and I called hypnobirthing practitioners. I called two of them, so I choose one of them and I bought the book, I read the book and finished the book before I went to the course. Then it was just like “okay this is what I want to do””.***IDI 5**

#### An easy process

The women perceived birthing as an effortless process with minimal or rather lack the risk of harm to mother and the unborn child. They were convinced of their ability for normal birth. Information from the resources on unassisted home birth further strengthen their confidence. They perceived birthing as a physiological process, and one woman explained the process of birthing philosophically as the blooming of a flower.

*“This (unassisted home birth) is a piece of cake. I gladly want to birth any day or every day because it is easy due to the techniques”***IDI 3**

*“Where is the danger? They talk about baby suffocating, drowning or having bacterial infection. There is no injury! Birthing is not an injury and your baby is not here to hurt you”***IDI 6**

*“You have to smile while giving birth. You have to breathe and you imagine blooming flower opening. The crowning of the head is actually clear rose is opening like that. And something beautiful and not to be scared...these videos, hospital birth and home birth that uses hypno-birthing techniques in which we saw how calm the women was, the wife, the mother was, and the husband have their roles”***IDI 3**

### Ensuring natural processes

The women refused any kind of medical interventions such as instrumental or invasive deliveries as they tried to experience as natural birth as possible. Investigation procedures such as monitoring with CTG and augmentation of labour were not preferred as well. All these medical interventions were perceived to increase unnecessary risks to their unborn child.

*“If you don’t disturb the birth, it’s safer that way. If you don’t disturb, the baby will come out fine. The body will most of the time keep the baby safe”***IDI 6**

*“The dilation drugs (referring to medications given increase uterine contraction) actually creates stress on the baby. It’s an artificial way of surges (referring to uterine contractions). That’s why the CTG shows the baby is in stress...There were like 14 procedures or something like that, done systematically upon caesarean section, all of which shocks the baby, who for the past 9 months is safe and sound.”***IDI 3**

*“I think it matters how your birth is and the birth itself will affect the child. How the baby comes out, by forceps or by vacuum. It actually affects the spine of the baby”***IDI 8**

*“...when you were in labour and then you had meconium, the doctor said its either vacuum or caesarean section. I don’t think it’s the most necessary at that time. Because I don’t feel there was any danger”***IDI 6**

### Theme 3: Expression of autonomy

#### Being in control

The women in this study expressed wanting to be the decision-maker regarding treatment and care, including the choice of birthing place. They believed medical personnel should not be the one who decides the next step of management during childbirth. Any interference by medical staff during the birthing process takes away the exclusivity and control of their birthing experience.

*“I’m able to give birth wherever I want... people respect and acknowledge the right that I have, and acknowledge the autonomy that I can choose wherever place that I want (to give birth)”***IDI 1**

*“...the freedom for the mothers is to deny or to refuse whatever that she is uncomfortable because all is about the mothers. The mother is battling with life and death battle. “So why are you (medical personnel) telling me what to do?””***IDI 3**

*“So, the rhythm of this labour, why is it important and how can you be involved in this labouring process. Because one of the time doctor will tell you “Okay, you come here, then you listen to me.” Hey, now it becomes the doctor’s birth not my birth”***IDI 6**

Before giving birth, some women in this study would develop birth plans which are used to convey their desired birth experience. It generally includes information such as where she wishes to give birth, who will attend a birth, and what forms of medical intervention and pain relief will be used. Their experience of healthcare providers rejecting their birth plan, specifically on home delivery was one of the strong pushing factors to divert against hospital delivery.

“*We discussed the birth plan. But it was not well accepted. You know the first thing doctor said like “Oh! This is very western.” That was his comment. And then like “Oh, we don’t know about this”. Things like that.”***IDI 6**

*“He (the doctor) just look at my birth plan and said “okay.. alright,.” It’s like very sarcastic and very like “yeah, right whatever.” You know. So, when he said “Yeah okay I’ll sign it.” But there are a few things he put like a question mark. Like can I move about (during labour) -he put question mark. I could sense that he is not going to change his ways. I found out that he never not did episiotomies. Which is scary. It is a standard procedure for him.”***IDI 8**

Some expressed that by giving the doctors permission to perform intimate physical examinations for example, vaginal examination equates to losing control of their own body. This led to a fear of giving birth in the hospital as they felt their rights were violated once they were in the labour room.

*“..once when I am in the hospital, they are going to do something that even I won’t do to myself. For example vagina examination. Your husband won’t do to you, even I won’t do it to myself. But, you give up your autonomy to this stranger.”***IDI 1**

*“... I didn’t have to be checked all the time (when giving birth at home). Somebody putting their fingers in me and all that.”***IDI 3**

#### Empowered

Generally, the women felt that giving birth at home gave them a sense of empowerment. They were able to do normal activities at home during labour, unlike in the hospital where they would be confined to bed for foetal monitoring. They believed that being in control of their body will eliminate fear, thus making birth safer as mothers were more in tune to what was happening in and around them.

*“It gives me some kind of empowerment. I was able to go about, go upstairs, downstairs, drink water and watch television. I was being relaxed at home. Doing normal things in between the contractions”***IDI 3**

*“Eliminate fear and then the women will have confidence. They feel empowered and are more in tuned with their body. That will make birth safer.”***IDI 6**

The women described the birthing process to be partly driven by their feeling of security, being in control of the birthing process and knowing exactly what to do to ease themselves. These feelings combined made them confident in giving birth unassisted at home.

*“I knew what was going with my body, I’m not insecure or afraid because I know what was going on and I know what it takes to relief myself. All I had to do was to do deep breathing and my husband had to do that light massage to release the hormone***” IDI 5**

*“I really wanted to give birth at home.. I will do it myself. Maryam (*Mary, Mother of Jesus) *(peace be upon her) did it herself. I’m going to birth myself...whether I’m at home, in the hospital, in the car, on a tree.. I know what my body does.”***IDI 1**

### Theme 4: Faith

Women proceeded with unassisted home birth as they have confidence in their ability to continue with the delivery of the newborn naturally as they submit totally to any outcome determined by the Almighty.

#### Self-trust

The women allowed their bodily instincts to lead the birthing process with a trust that an undisturbed birth would most likely to confer into a positive outcome. Total belief and submitting their body and mind wholeheartedly to God gave a sense of calmness to the women.

*“My level of confidence to God, at that time, I knew you (referring to God) created me to give birth naturally because you (referring to God) are the Fairest, Most Loving. You are not subjecting me to the pain. Because of that, I am relaxed about giving birth at home.”***IDI 3**

*“It’s a very spiritual experience. There are many people who felt during the birth … spiritual presence or whatever. Feel connected”***IDI 6**

#### Fatalism

These women believed their pregnancy itself was a gift from God, and God also drove the process of birthing. This stance provided the courage to overcome the fear of labour as they surrendered to any outcome that might occur.

*“You see, that’s why our five (National Principles of Malaysia)... The first one is Trust in God, right? Basically that helps to remove fear because when you remove fear you can hear your instinct clearer. Because when you are scared your body tensed up. Then you, actually create the complication*” **IDI 6**

*“It’s so magical because I surrendered to God. ... Not doctors, not nurses, not my mother or mother-in-law, or my dad. Ultimately it’s you and God... between life and death”***IDI 3**

These women believed that by total submission to God, they do not need medical advice, and they are fully responsible for their decisions. With this firm belief, they are free to choose their birthplace and whatever consequences that may happen is because of fate.

*“Fate and destiny isn’t it? There will be risks wherever you give birth, either in hospital or at home.”***IDI 2**

*“I’m a perfect creation of my God and why do I doubt my creation? We (humans) are perfect. We can give birth; we can be pregnant.”***IDI 12**

One woman reported a critical situation when her baby was born, and there was suspicion of a complication from the birth process. She reflected the miracle of total submission to God even though her baby was born lifeless with the umbilical cord encircling around the neck.

*“You know the transition of the “spirit” coming in …*. *because when my baby was born she did not cry. There was no sign of her at all. This is scary ... and the next thing you know...she cried right after the adzan (call for prayer) and you’ll be in awe”***IDI 12**

## Discussion

Birth should be a positive life-changing experience, but it is also a significant life event that could have profound effects on a woman’s physiological and psychological wellbeing. This study seeks to find reasons for women to choose unassisted birth at home. Not many women had chosen to do this, and among those who had, most were quite reserved to discuss the topic outside of their inner-circle.

In this study, none of the women was pregnant at the time during the period of the interview, and the women had unassisted homebirth a few years ago. Only one woman had unassisted home birth during her first pregnancy. The other women only resorted to unassisted home birth following their previous hospital births. Hence, the mean age of women in this study is comparatively older compared to the local, national mean age of childbearing, at 30.74 years old [28]. Similarly, their tertiary educational attainment is higher compared to the average Malaysian of 25 years and above [29].

The four themes that emerged from the interviews strongly relate to the needs of these mothers; having a beautiful and natural birthing experience while still maintaining the autonomy to decide what they perceived best for them. Our study concurs with a review that reported about first-time mothers who wished that their needs to be respected during labour, ‘to feel involved in the care’ and to have their partners supporting them in their journey [30]. The setup of a hospital delivery environment is very clinical, and the obstetric team are more focussed in the care of their patients. These women do not want to be in the hospital as they do not have pathology or disease that needs a clinical setting governed by strict medical, regulatory rules and institutional guidelines [6, 13]. The need for a comfortable and private environment has been expressed in a few studies worldwide [31, 32].

In this study, privacy is one of the main reasons for mothers to choose unassisted birth. The hospital environment was perceived as exposing the mothers to strangers, namely hospital staff. However, maintaining privacy is a challenge in any hospital settings [33]. The medical staff needs to change at every shift to ensure a safe environment by preventing tiredness and human error; thus, a mother with more than 12 h of labour experience could be attended to by three different teams of nurses [34, 35]. Deficiency in effective communication and insufficient delivery suite added to the stressor [6].

The United Nation Global Strategy for Women’s, Children’s and Adolescents’ Health acknowledges the need to address not only the clinical requirements for a safe labour and childbirth but also the psychological and emotional needs of the women involved [36]. The women-centred philosophy and human-rights approach allows this to be done and would ultimately have a positive impact on women’s experience of giving birth [36]. Despite the introduction of mother-friendly care in all baby-friendly hospital in Malaysia in 2012, much can be done to improve the environment in the hospitals to make them more comfortable and mindful of mothers’ need for privacy, without compromising their safety.

To materialise the concept of maintaining a good psychological and emotional wellbeing of mothers, one needs an adequate number of trained, skilled health care personnel. At the current state, midwives (or nurses with post-basic midwifery training) in the Malaysian public healthcare setting are overwhelmed with their clinical duties. The 2010 local statistics highlighted that there are about 400,000 deliveries in Malaysian public hospitals, and they are conducting about 200 births per person [37]. The recommended delivery to midwife ratio is 35:13, and this indicated that the requirements are not being adequately met. Therefore, we must ensure an adequate number of trained midwives or nurses with post-basic midwifery training to provide more women-friendly services in the hospitals (public and private) and expand their services for assisted low-risk home births in Malaysia.

Quaternary prevention is a concept that emphasizes on “first, do no harm”. It is developed by a Belgian family physician, Marc Jamoulle. It is a set of activities employed to identify people who are at risk of over-medicalisation and to reduce unnecessary or excessive interventions to minimise iatrogenicity [38]. This concept is also important to humanise childbirth and combatting obstetric violence whilst not forgetting scientific evidence-based practice. One of the ways to implement this is to perform clinical audits on maternity facilities [39]. Although clinical audits are already in place in the current public healthcare system in Malaysia, it would be valuable to update this practice to improve healthcare services, especially in maternal healthcare.

The medicalization of birth has created a division between “natural” and “medical/hospital” birth. Antenatal and intrapartum care has improved tremendously over the past decade, and this has resulted in the increasing ability to recognise and address possible complications arising from pregnancy and birth. Some women in our study strongly believed that birthing is easy, safe, and God had created their body capable of giving birth naturally and independently. Perceived competency could be a motivator to their decision making for unassisted home birthing [40–42]. Having a good “body knowledge” and about birth itself helped these women to negotiate the birth process without medical assistance calmly [43].

The technology of modern medicine is synonymous to images of drug-induced births and instrumentation during delivery, which leads to fear of the interventions being given during the hospital birthing process. The “no intervention” during home birth may contribute to the perception that unassisted home birth is safer than the hospital [44]. A local study also revealed a high percentage (40.5%) of women having preferred a natural birthing, which to them was defined as “a system of managing childbirth in which the mother receives preparatory education in order to remain conscious and assist in delivery with minimal or no use of drugs or anaesthetics” [45].

The women in our study had a few misconceptions regarding interventions during birthing, which were perceived as unnecessary risks to them and their unborn child. In particular, the medical interventions to hasten delivery such as augmentation of labour and assisted delivery when there is a sign of foetal distress. A meta-analysis comparing outcomes of women who had planned home births and planned hospital births in 2010 showed a doubling of the neonatal mortality rate in planned home births (regardless of assistance) [46]. The women who had planned home births were of similar and often lower obstetric risks than those planning hospital births. Although the women with planned home births experienced significantly fewer medical interventions, infections, lacerations and retained placenta; it is alarming to see the high neonatal mortality rate in that group. Intrapartum asphyxia was implicated as a possible cause of neonatal death in planned home births [46]. There may be other unrecognised factors that contributed to the high neonatal mortality rate found in that meta-analysis. These risks will be higher in the absence of trained healthcare personnel and appropriate equipment to handle any obstetrics emergencies during labour.

On the other hand, a systematic review and meta-analysis of planned place of birth among low-risk pregnancies in high incomes countries showed no significant differences in early neonatal death between planned births at home (assisted births) and in hospital [47]. Another meta-analysis in 2014 from the Netherlands also showed no increased risk of adverse perinatal outcomes for planned home births among low-risk women [48]. However, the results may only apply to countries where low-risk home births are planned, assisted and well-integrated into the maternity care system. The women in our study have variable obstetric risks including grand multiparous, previous caesarean sections and primigravida. Therefore, higher complication rates may be observed in women with the above risks, in which home births may not be appropriate.

Being involved in decision making and wanting to be in control in their birth plans gives the women in this study a sense of control which concurs with previous studies [3, 40, 49]. They were not able to always be in control when birthing in the hospital. There is a conflict between the doctors’ interest to keep the women and their baby safe versus the women’s wish to deliver without any intervention, without understanding the risk of their decision. This is perhaps why true autonomy is not feasible in the hospital. Giving birth unassisted at home abled them to give birth according to their birth plans and wishes. Many were traumatised by the experience of vaginal examinations and vacuum-assisted births, and these were equated to losing control during the birthing process [49]. Being in control of one’s body is important to eliminate fear during birthing [50]. Women have described the sense of empowerment after giving birth unassisted at home [41, 43]. This sense of empowerment motivates them further to pursue home birth. Empowerment and self-confidence allowed the mothers to relax and experience their births with the clear understanding that their input would be listened to and honoured whenever possible [51].

Shared decision making (SDM) is an important element in healthcare as it empowers the patient in the management of their condition and promotes good doctor-patient relationships. SDM is already a common practice in Malaysian healthcare settings in general. In the SDM model, doctors use their professional expertise to present and explain options to patients, and patients use this information to develop preferences based on their values and priorities. Both parties then negotiate a final shared decision. This model assumes that the patient can participate in decision making; however, doctors will need to know when good decisions are not being made and how to intervene appropriately [52]. It is a challenge to apply the SDM model while facing an obstetric emergency; such as foetal distress or cord prolapse as there may be limited time to discuss other options with the woman in labour [53].

In our study, women were seen to be surrendering themselves in total to God. Compared to previous studies, this emerging theme of “faith” plays a strong role in their decision making, and it made them choose unassisted home birthing as they strongly believe that God will assist with the birthing process, even if it happens to be a difficult one. This is similar to another report that patients’ belief in God and prayer were so strong that they believe they no longer need conventional medical care [54]. The women have a strong belief that their birthing process would be guided by God’s will, and they submit themselves totally without wanting any intervention. Having faith was closely linked to receiving guidance, protection and rewards from God [55]. There are women who also believed that God would reduce their labour pain when they prayed [56]. These findings were also echoed among women in our study.

It is important for health professionals to acknowledge women’s beliefs and at the same time, to treat the women holistically, regardless of their religious beliefs. These women made their decision to birth at home without medical assistance, with the strong belief that God will be there to help them; a concept of “*tawakkal*”. However, according to the teachings of Islam (the official religion in Malaysia), apart from having the concept of “*tawakkal*”, there is also the need to seek professional medical help as part of their effort to ensure a safe pregnancy and birth [57]. The office of Mufti of the Federal Territory of Malaysia has declared the practice of delivering a baby without the observation of a doctor or trained medical personnel in Malaysia is deemed to be unacceptable by Islamic laws [58]. This is based on religious hadiths that support leaving issues pertaining to a topic to those who are experts in that field, the inappropriateness to directly infer the delivery experience of a mother to a prophet to the delivery of other mothers and Islam’s view on doing no harm [58].

Some women in this study still receive their antenatal care from either the public or private healthcare facilities and their reason was to ensure that they are free of any kinds of diseases like gestational diabetes or hypertension in pregnancy that may interfere or impair their pregnancy and its outcome. This highlights the crucial role antenatal care may have in identifying potential women who may plan to have unassisted home births. The antenatal team may be able to intervene unassisted home birth plans by providing individualized care with good interpersonal and clinical skills in a respectful manner at every contact. Obstetric risk stratification and availability to have trained and skilled healthcare personnel are crucial to determine suitability to have assisted home births.

The strength of this study is this is the first paper to explore the phenomena of unassisted home birth in Malaysia, a middle-income country with a good and accessible health system. This study included mothers who had personal experience of unassisted home birth, and they are of variable backgrounds (mothers, home birth advocators and natural birth advocators). However, our limitations are that only women who had a favourable outcome and positive unassisted home birthing experience participated in this study. Thus, their reasons for choosing unassisted home birth discussed here may not represent other women who have given birth at home. The authors did not analyse the maternal age at the time of their unassisted birth; thus the women in this study may falsely be labelled as “older mothers”.

## Conclusion

This qualitative study observed that the need to keep the birthing comfortable and private, the belief that birthing itself is a natural and safe process, wanting autonomy and faith as reasons for choosing unassisted home birth. The findings highlighted that the current maternal healthcare is less appealing to some women. More resources are needed in creating a more personalised birthing within the hospital setting without compromising safety matters. The current issue of lack of workforce (trained midwives) needs to be addressed so that we can provide more women-friendly services in the hospitals as well as providing services for low risk assisted home births.

## Data Availability

The data that support the findings of this study are available on request from the corresponding author JS. The data are not publicly available due to information that could compromise research participant privacy and consent.

## Abbreviations

IDI: In-depth interview
SDG: Sustainable Development Goals
SDM: Shared decision making
TBA: Traditional birth attendants
WHO: World Health Organization
